# COVID-19 Exacerbates Neurovascular Uncoupling and Contributes to Endothelial Dysfunction in Patients with Mild Cognitive Impairment

**DOI:** 10.3390/biom14121621

**Published:** 2024-12-18

**Authors:** Cameron D. Owens, Camila B. Pinto, Zsofia Szarvas, Mihaly Muranyi, Ana Clara da C. Pinaffi-Langley, Anna Peterfi, Peter Mukli, Sam Detwiler, Lauren Olay, Zalan Kaposzta, Kenneth Smith, Angelia C. Kirkpatrick, Faddi Saleh Velez, Stefano Tarantini, Anna Csiszar, Zoltan I. Ungvari, Calin I. Prodan, Andriy Yabluchanskiy

**Affiliations:** 1 Oklahoma Center for Geroscience and Healthy Brain Aging, University of Oklahoma Health Sciences, Oklahoma City, OK 73117, USA; cameron-owens@ouhsc.edu (C.D.O.); camila-boninpinto@ouhsc.edu (C.B.P.); zsofia-szarvas@ouhsc.edu (Z.S.); mihaly-muranyi@ouhsc.edu (M.M.); anaclara-dacostapinaffilangley@ouhsc.edu (A.C.d.C.P.-L.); anna-peterfi@ouhsc.edu (A.P.); peter-mukli@ouhsc.edu (P.M.); sam-detwiler@ouhsc.edu (S.D.); lauren-olay@ouhsc.edu (L.O.); zalan-kaposzta@ouhsc.edu (Z.K.); stefano-tarantini@ouhsc.edu (S.T.); anna-csiszar@ouhsc.edu (A.C.); zoltan-ungvari@ouhsc.edu (Z.I.U.); 2 Vascular Cognitive Impairment and Neurodegeneration Program, Department of Neurosurgery, University of Oklahoma Health Sciences, Oklahoma City, OK 73104, USA; 3 International Training Program in Geroscience, Doctoral School of Basic and Translational Medicine/Institute of Preventive Medicine and Public Health, Semmelweis University, 1089 Budapest, Hungary; 4 Department of Nutritional Sciences, College of Allied Health, University of Oklahoma Health Sciences, Oklahoma City, OK 73117, USA; 5 Department of Physiology, Faculty of Medicine, Semmelweis University, 1087 Budapest, Hungary; 6 Oklahoma Medical Research Foundation, Oklahoma City, OK 73104, USA; ken-smith@omrf.org; 7 Veterans Affairs Medical Center, Oklahoma City, OK 73104, USA; angelia-kirkpatrick@ouhsc.edu (A.C.K.); calin-prodan@ouhsc.edu (C.I.P.); 8 Cardiovascular Disease Section, Department of Medicine, University of Oklahoma Health Sciences, Oklahoma City, OK 73117, USA; 9 Department of Neurology, University of Oklahoma Health Sciences, Oklahoma City, OK 73104, USA; faddi-salehvelez@ouhsc.edu; 10 Department of Health Promotion Sciences, College of Public Health, University of Oklahoma Health Sciences, Oklahoma City, OK 73104, USA; 11 Peggy and Charles Stephenson Cancer Center, University of Oklahoma Health Sciences Center, Oklahoma City, OK 73104, USA

**Keywords:** mild cognitive impairment, neurovascular coupling, COVID-19, endothelial function, flow-mediated dilation

## Abstract

Mild cognitive impairment (MCI) affects nearly 20% of older adults worldwide, with no targetable interventions for prevention. COVID-19 adversely affects cognition, with >70% of older adults with Long COVID presenting with cognitive complaints. Neurovascular coupling (NVC), an essential mechanism of cognitive function, declines with aging and is further attenuated in neurocognitive disorders. The effect of COVID-19 on NVC responses has yet to be addressed in older adults who are vulnerable to dementia progression. Participants with MCI and a history of COVID-19 (COV+, N = 31) and MCI participants with no history of infection (COV− N = 11) participated in this cross-sectional study to determine if COVID-19 affects cerebrocortical NVC responses and vascular function. Functional near-infrared spectroscopy was used to measure cerebrocortical NVC responses, and endothelial function was assessed via insonation of the brachial artery during a flow-mediated dilation protocol. NVC responses were elicited by the working memory *n*-back paradigm. NVC in the left dorsolateral prefrontal cortex and endothelial function was decreased in the COV+ group compared to the COV− group. These data provide mechanistic insight into how COVID-19 may exacerbate long-term cognitive sequela seen in older adults, highlighting the urgent need for further research and clinical trials to explore novel therapeutic interventions aimed at preserving/restoring NVC.

## 1. Introduction

Mild cognitive impairment (MCI) is a prevalent age-related condition affecting nearly 20% of older adults worldwide [1]. Fluid cognitive abilities (cognition not dependent on past learned experience) decline as we age [2], with further impairment in patients with MCI [3]. While research suggests that targeted lifestyle changes may enhance cognitive function, stabilize the condition, or, in some cases, even reverse it to normal cognitive functioning, there is no definitive evidence that interventions can fully prevent the transition from healthy aging to MCI [4]. Moreover, MCI is a prodromal stage of dementia, a progressive condition with no curative therapy, regardless of etiology. Due to the varying etiologies of MCI, such as Alzheimer’s disease and cerebrovascular disease, identifying a common, targetable mechanism to prevent further cognitive decline is crucial [5,6,7,8].

Neurovascular coupling (NVC) is a critical mechanism of cognitive performance, playing a vital role in cognitive processing throughout the lifespan [6,9,10,11]. NVC functions as a feed-forward mechanism initiated by local neuronal activity to increase blood flow that is dependent on the cerebrovascular endothelium [6,12,13,14]. This increase in blood flow provides nutrients to neurons and the washout of toxic metabolic by-products [12]. Preclinical evidence indicates a causal relationship between NVC and cognitive performance in aging and age-related neurocognitive disorders [11,15]. Importantly, anti-aging interventions rescue the NVC response [13,14] and improve cognitive performance in mice [16], highlighting NVC as a critical target for future human clinical trials. To enhance the understanding of the NVC response in humans, we determined that NVC responses were positively associated with increased cognitive performance and were impaired in older adults compared to young adults [2] and in individuals with MCI compared to age-matched controls [3], signifying NVC as a crucial mechanism for maintaining cognitive abilities as we age [6,11].

Besides that, endothelial function is a key determinant of vascular health and has significant implications for both peripheral and cerebral circulatory systems. The assessment of endothelial function using the flow-mediated dilation (FMD) protocol, which involves the insonation of the brachial artery, is a widely used non-invasive method to evaluate macrovascular endothelial responses. Recent research has highlighted the potential of FMD to serve as a surrogate marker for generalized endothelial dysfunction and cognitive impairment [16,17]. The link between peripheral vascular health and NVC is underscored by findings from our group and others (red), which demonstrated that age-associated declines in peripheral vascular function, as measured by FMD and arterial stiffness indices, correlate strongly with cognitive impairment. These deficits align with deteriorations in cerebral microcirculatory function and NVC, emphasizing the systemic nature of vascular dysfunctions.

COVID-19, caused by the SARS-CoV-2 virus, has emerged as a significant public health crisis with a widespread impact on various bodily systems, including the nervous and vascular systems [12]. This virus has particularly severe consequences for older adults, exacerbating pre-existing conditions and contributing to new health challenges, such as cognitive impairment and endothelial dysfunction. Older adults are particularly vulnerable to the neurological and cognitive effects of COVID-19 [18,19], which can exacerbate pre-existing conditions like MCI [20]. SARS-CoV-2 exhibits endothelial trophism [21,22,23,24], and acute and long-term effects of SARS-CoV-2 infection have been associated with endothelial dysfunction and other vascular abnormalities [24,25,26,27,28,29,30,31,32,33,34,35,36,37]. Recent findings determined COVID-19 as a causal and contributing factor to cerebral small vessel disease (CSVD) pathology in older adults [38]. Importantly, CSVD is a significant contributor to cognitive impairment worldwide [39,40] and is present in the majority of the oldest-old adults [41]. While the vascular and cognitive impacts of both acute and Long COVID-19 are well-documented, the role of the NVC response, which primarily relies on cerebral small vessels and endothelial function in COVID-19-induced cognitive dysfunction, remains undetermined. Understanding this connection is crucial for developing targeted interventions to mitigate the cognitive impact of COVID-19 in older populations.

In this cross-sectional study, we hypothesized that a history of COVID-19 would further disrupt NVC responses in patients with MCI compared to those without a history of COVID-19. Secondarily, we aimed to determine differences in peripheral endothelial function. We and others have previously shown that peripheral endothelial function predicts cognitive impairment [42,43]. As COVID-19 is a systemic disease [44], we hypothesized that peripheral endothelial function would be impaired in patients with MCI with a history of COVID-19 (COV+) compared to those with no history of infection (COV−).

## 2. Materials and Methods

### 2.1. COVID-19 Severity

All participants in the COV+ group were classified as having mild COVID-19 based on their self-reported symptoms, following CDC guidelines (i.e., no patients requiring hospital admission and supplemental oxygen). This classification included participants who reported no symptoms but tested positive through blood tests. None of the participants required hospital admission or supplemental oxygen. One participant sought care at the emergency department due to fatigue but did not report respiratory symptoms or require hospitalization or supplemental oxygen.

### 2.2. Study Participants and Measurement Groups

All participants enrolled in the study signed the institutionally approved informed consent. Community-dwelling older adults with MCI were recruited as a part of a large prospective longitudinal study to assess COVID-19’s effect on progression to dementia. Participants in this study were clinically diagnosed with MCI based on comprehensive clinical evaluations, which included cognitive testing, symptom assessment, and patient history. While MCI is a heterogeneous condition that can sometimes remain stable or even revert to normal cognition under certain conditions, such as with lifestyle interventions, the participants included here met criteria for MCI based on real-world clinical judgment and were not classified solely according to research-specific definitions.

In the current study, we conducted a comprehensive cross-sectional analysis comparing baseline visit data between participants diagnosed with MCI who have a history of COVID-19 (COV+) and participants with MCI who have no history of COVID-19 (COV−). Inclusion criteria consisted of age ≥ 65 years old, Clinical Dementia Rating equal to 0.5, and/or 30-point Montreal Cognitive Assessment (MoCA) < 26 and ≥19. For participants where other clinical cognitive assessments were performed (telephone MoCA, MoCA for the blind, mini mental state evaluation), published meta-analysis conversion charts were used to convert these scores to 30-point MoCA equivalents [45,46]. All participants abstained from consuming caffeine on the day of measurement.

We excluded participants with the following conditions: (1) chronic uncontrolled medical conditions (e.g., hypertension and type 2 diabetes); (2) chronic complications from prior stroke (e.g., aphasia and hemiparesis); (3) significant neurodegenerative diseases (such as Parkinson’s disease, multiple sclerosis, and amyotrophic lateral sclerosis, since these conditions may independently influence cognitive function); and (4) any medical, psychiatric, or medication-related problems that in the opinion of the research and clinical neurology team would render participants unable to participate in the study.

**Figure A1:**
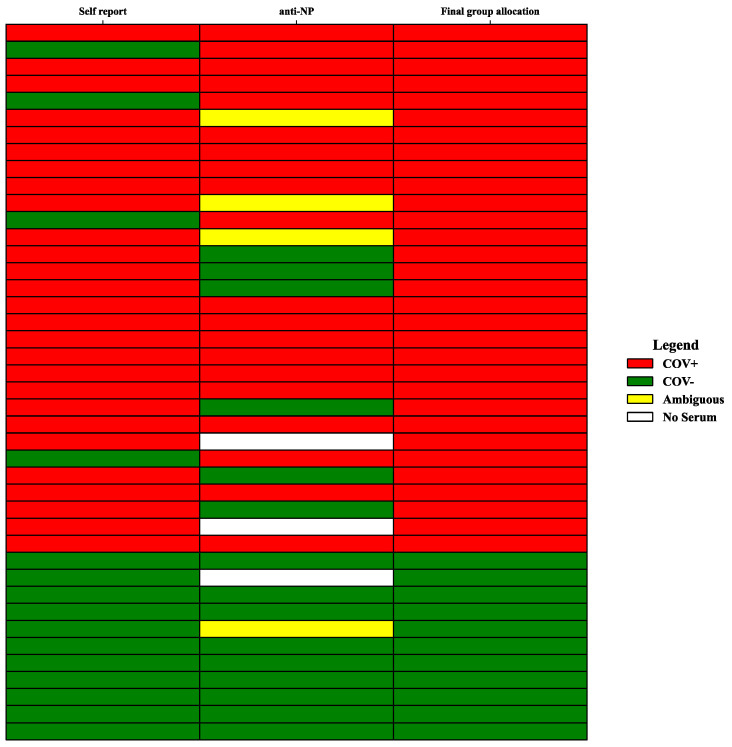
**Final COVID-19 group allocation.** Self-report (**left**) of participants COVID-19 history (e.g., PCR, antigen, and serology tests), COV+ *n* = 27, COV− *n* = 15. Anti-nucleocapsid protein (NP) test results (**middle**) of participants who were able to complete blood draw (*n* = 39). Positive control value reached an optical density (OD) of 3.0; the ambiguous cut-off was 0.94 (average of three sample runs plus three SD), with COV+ values above and COV− below the ambiguous cut-off. Final group allocation (**right**) was determined as follows: self-report COV+ with anti-NP COV+, final group allocation COV+; self-report COV+ with anti-NP COV−, final group allocation COV+; self-report COV− with anti-NP COV+, final group allocation COV+; self-report COV− with anti-NP COV−, final group allocation COV−.

To confirm group classification (COV+ or COV−), participants who provided blood samples (*n* = 39 of 42) underwent a nucleocapsid antibody test (anti-NP) using the SARS-CoV-2 B.1.1.529 [Omicron] nucleocapsid protein assay (Sino Biological, Wayne, PA, USA), along with self-report data. Participants with documented COVID-19 were included in COV+ group irrespective of anti-NP, and participants who self-reported no COVID-19 history but had a positive anti-NP were included in the COV+ group. For participants without blood samples (*n* = 3), the classification was based solely on self-reported COVID-19 history, as shown in the final group allocation column (rightmost column in Figure A1).

### 2.3. Blood Processing, Storage, and Nucleocapsid Antibody Analysis

Blood was collected from participants in two 10 mL BD Vacutainer serum tubes (BD, Franklin Lakes, NJ, USA) and processed for serum by centrifugation for 10 min at 1600 RPM. Serum was stored in 30 μL aliquots in −80 °C freezer for anti-NP analysis. For the determination of anti-NP positivity by ELISA, high-binding ELISA plates (Corning, Costar 3369, Corning, NY, USA) were coated with 0.2 µg/well of SARS-CoV-2 B.1.1.529 [Omicron] Nucleocapsid Protein (Sino Biological, Wayne, PA, USA) overnight at 4 °C. For all subsequent washes, plates were washed four times with PBS-Tween (0.05% Tween). After coating overnight, plates were washed and then blocked with 75 μL/well 0.1% BSA in PBS for 1 h at room temperature (RT). The plates were again washed, and samples were loaded onto the plates. A strongly positive control serum and patient samples were diluted 1:200, and 60 μL/well was loaded in duplicate. The samples were incubated on the plates for two hours at RT and then washed. A total of 75 μL/well HRP-conjugated goat anti-human IgG (Jackson Immunoresearch, West Grove, PA, USA) diluted 1:1000 was then added and incubated for 1 h at 37 °C. The plates were washed one final time, and 100 μL/well of Super Aqua Blue ELISA substrate (Invitrogen, Eugene, OR, USA) was added. The OD405 was read until the positive control sample reached an OD of 3.0. All samples were run on at least three different experiments on three different days. To determine a cutoff value of positivity, 40 samples from either pre-2019 or samples from an mRNA-vaccinated, non-infected COVID-19 cohort were run using this protocol. The OD405s for these samples were averaged, and three standard deviations were added to this value for a numerical cutoff OD of 0.94.

### 2.4. Neurovascular Coupling Protocol, Preprocessing and Analysis

NVC responses were determined by continuous changes in cerebrocortical oxy-hemoglobin concentration [HbO] via functional near-infrared spectroscopy (fNIRS), as described [47]. In brief, participants wore a 128-port head cap (Easycap GmbH, Woerthsee-Etterschlag, Germany) with 16 sources and 16 photodetectors (NIRx Medical Technologies LLC, Glen Head, NY, USA) that covered the frontal cortex according to the international 10–20 system. Light sources were emitted in the near-infrared range (760 nm and 850 nm) to detect relative changes in deoxyhemoglobin and HbO. Forty-eight source–detector pairs were separated by 3 cm for sufficient penetration of the cerebral cortex at a depth of ~1.5 cm. All fNIRS signals were recorded with a 3.9 Hz sampling frequency.

The n-back working memory paradigm reliably elicits changes in cerebrocortical oxy-hemoglobin concentration ([HbO]) by engaging task-relevant neural circuits, particularly in the prefrontal cortex [2]. As cognitive demands increase across 0-back, 1-back, and 2-back conditions, neuronal activity rises, driving localized increases in cerebral blood flow and [HbO] through neurovascular coupling. The paradigm systematically manipulates cognitive load, with the 2-back condition imposing the greatest working memory and executive function demands. These graded hemodynamic responses have been validated in numerous studies, demonstrating robust and reproducible [HbO] increases in task-relevant regions using functional near-infrared spectroscopy (fNIRS) [48,49,50,51]. This paradigm is thus a well-established method for eliciting and measuring cerebrocortical activation and changes in [HbO].

Briefly, participants were seated in front of a computer monitor and were instructed to click the left mouse button when the stimulus target appeared. Three conditions were administered, as follows: 0-back (0b_1) was administered first, where participants were instructed to click the left mouse button when the letter “**W**” appeared on the screen. During the 1-back (1b) condition, participants were instructed to click the left mouse button when repeated letters appeared (i.e., “A” “**A**”). During the 2-back (2b) condition—the most cognitively challenging task—participants were instructed to click the left mouse button when a letter that is the same as the 1–2-back was presented on the screen (i.e., “A” “C” “**A**”). A second 0 back (0b_2) was presented before the 2-back condition for a total of four blocks of cognitive tasks (~72 s per block).

Preprocessing of fNIRS data was implemented in Matlab 2023b (Mathworks, Natick, MA, USA) using custom scripts written by the authors, executed in a pipeline employing the Brain AnalyzIR toolbox (commit 46c645d) [52]. Motion and physiological artifacts not arising from brain activity were removed as previously described [47]. For preprocessed optical density conversion to [HbO], we applied the modified Beer–Lambert law [53]. HbO was pre-whitened to reduce serially correlated effects from artifacts. Slow drifts were removed by discrete cosine transform-based high-pass filter (0.009 Hz). To assess NVC responses at the subject and group level, general linear model (GLM) approach was employed to generate specific regression coefficients (β), as previously described [54]. Left dorsolateral prefrontal cortex (LDLPFC) was an a priori region of interest due to recent findings of this area predicting cognitive impairment with high feature importance [2,3,9,12,38,47,54].

### 2.5. Peripheral Endothelial Function Protocol, Preprocessing, and Analysis

Endothelial function was determined from the brachial artery using the gold standard flow-mediated dilation (FMD) approach, as previously described [47]. Briefly, blood pressure was measured, and participants were instructed to rest supine for 20 min prior to brachial artery insonation. Following cuff placement distal to the antecubital fossa and ultrasound visualization of the vascular intima, one minute of baseline brachial artery diameter was recorded. Blood flow was occluded at 50 mmHg above systolic blood pressure for five minutes. After five minutes of occlusion, the cuff was released, and changes in brachial artery diameter were measured.

FMD% was calculated as previously described [47]. Briefly, continuous change of brachial artery diameter was sampled at 15 frames per second, and BrachialAnalyzer software (version 6.9.4) employed automatic edge detection to determine intimal boundaries for analysis. Cardiac cycle R-peaks were removed for all frames to eliminate the influence of the cardiac cycle prior to final diameter determination. To calculate the FMD response, relative maximal diameter change following occlusion is expressed in percentage: FMD% = [(Max diameter − average baseline diameter)/(average baseline diameter) × 100].

### 2.6. Statistical Analysis

Fisher’s exact test, parametric two-tailed unpaired *t*-tests, simple linear regression, GLM, and Mixed-Effects Models (MEM) were performed in Prism 10.0.2. Brain AnalyzIR was implemented in MATLAB R2023b for group-level analysis of NVC responses. All data presented are shown as mean ± SD unless stated otherwise. If Shapiro–Wilk test showed normal distribution (*p* > 0.05), parametric tests were conducted. For n-back accuracy (*d’*) and reaction time (RT) analysis, GLM and MEM were used, respectively, to handle nonparametric data and data with missing values. NVC data analysis was conducted as previously described [3]. For NVC group comparison, type 1 error was mitigated by setting significance at *q* < 0.05 after controlling for the false discovery rate using the Benjamini–Hochberg procedure. Heat map of participant final group allocation based on COVID-19 history self-report data and anti-NP test was conducted in Python 3. *d’* was calculated as previously described [2]. RTs were recorded in milliseconds to determine correct responses to *n*-back stimuli. To assess the relationship between MoCA values and cognitive performance, we employed simple linear regression to derive regression equations for *d’* and RT. These equations allowed us to predict each participant’s accuracy and reaction time (Y) based on their MoCA scores (X). We then calculated the residuals by subtracting the observed accuracy (*_m_d’*) or reaction time (*_m_*RT) from the predicted values. These residuals represent cognitive performance independent of baseline cognitive status, enabling us to determine the influence of COVID-19 on cognitive performance.

## 3. Results

### 3.1. Study Participants

The characteristics of the study groups and baseline physiological data COV+ participants (N = 31, 74.16 ± 4.82 years of age) and COV−participants (N = 11, 73.18 ± 5.76 years of age, *p* = 0.5850) are summarized in Table 1. COV+ and COV− groups did not differ in sex; race; ethnicity; education level; blood pressure; heart rate; and cardiovascular, psychological, and cerebrovascular diseases. COV− patients had worse clinical cognitive scores (Montreal Cognitive Assessment [MoCA]) at baseline compared to COV+ patients (COV+, 24.35 ± 2.537; COV−, 22.55 ± 2.296, *p* = 0.0440). Therefore, we used adjusted MoCA scores (see Section 2.6) for cognitive testing to determine cognitive performance irrespective of baseline cognitive status that was not attributable to COVID-19.

### 3.2. Neurovascular Coupling Is Impaired During Increased Cognitive Load in MCI Participants with a History of COVID-19

**Figure 1:**
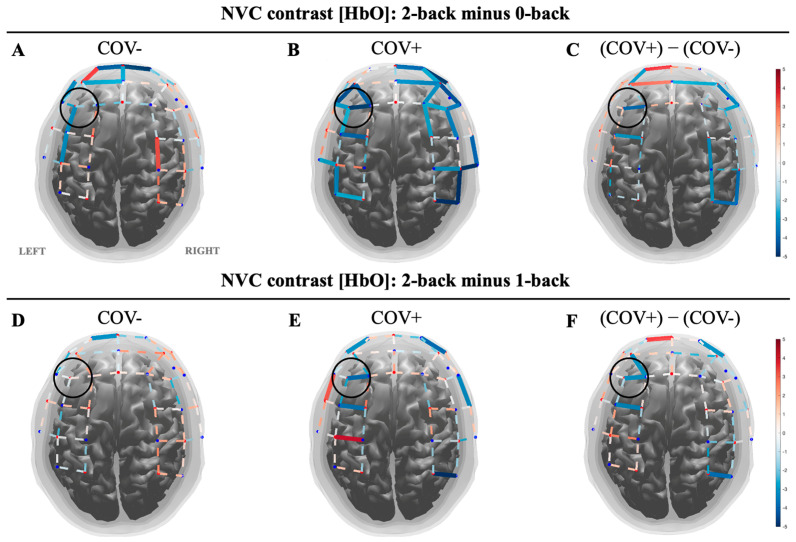
Neurovascular uncoupling is exacerbated in the left dorsolateral prefrontal cortex during tasks with increased cognitive demand in patients with MCI and a history of COVID-19. Within group statistical comparison shows decreased LDLPFC NVC for COV− in both contrasts assessing the most cognitively challenging task compared to less demanding tasks (**B**,**E**). The COV− group showed decreased LDLPFC NVC responses in 2b minus 0b (**A**) and no change in 2b minus 1b (**D**). Between group comparison determined that COV+ NVC responses were decreased in the LDLPFC during the most cognitively challenging task relative to tasks that were less difficult compared to the COV− group. (**C**,**F**). The black circle highlights the LDLPF. Statistically significant increases and decreases in [HbO] are shown in red and blue solid lines, respectively, with colors corresponding to t-statistics thresholded by q < 0.05 after false discovery rate (FDR) correction.

**Figure A2:**
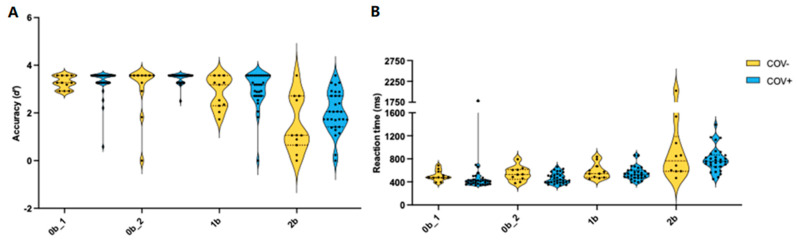
**Uncorrected working memory cognitive performance of participants with MCI.** Distribution of MCI participant accuracy (*d’*) during the n-back working memory task in COV+ (blue) and COV− (yellow) groups (**A**). Distribution of MCI participant reaction time during the n-back working memory task in COV+ and COV− groups (**B**). Accuracy was calculated as described (see main text). Reaction time data were obtained from correct responses only. Violin plot’s solid line and dashed lines represent median and 25th and 75th percentiles, respectively.

To determine the NVC responses to cognitively challenging tasks, the contrasts 2b-0b and 2b-1b were used for within- and between-group comparisons. The black circle highlights the left dorsolateral prefrontal cortex, a region strongly implicated in working memory and cognitive control. Statistically significant increases and decreases in [HbO] are shown in red and blue solid lines, respectively, with colors corresponding to t-statistics thresholded by q < 0.05 after false discovery rate (FDR) correction. Within-group statistical comparison shows decreased LDLPFC NVC for COV+ in both contrasts assessing the most cognitively challenging task compared to less demanding tasks (B, E). The COV− group showed decreased LDLPFC NVC responses in 2b-0b (A) and no change in 2b-1b (D). The between-group comparison determined that COV+ NVC responses were decreased in the LDLPFC during the most cognitively challenging task relative to tasks that were less difficult compared to the COV− group Figure 1C,F. We examined sex as a biological variable to determine if there were any differences between male and female subjects. Our analysis revealed no significant differences between the sexes. Additionally, age, sex, and education were accounted for in the supplementary Figure A2 NVC analysis to partially account for the influence of sex on the outcomes of this study.

### 3.3. Cognition Performance Is Unaffected Between MCI Groups

**Figure 2:**
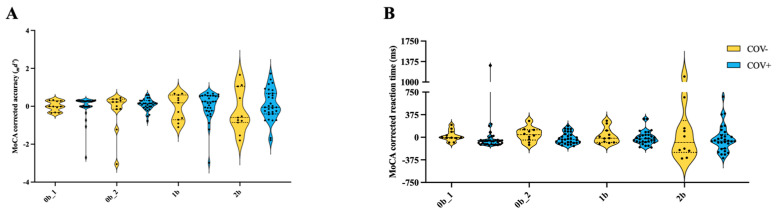
Cognitive performance is unaltered in MCI participants with a history of COVID-19. Panel (**A**) shows the MoCA-corrected cognitive performance (*_m_d’*), and Panel (**B**) shows the MoCA-corrected reaction time (*_m_*RT) quantified as the residuals (observed - predicted performance). There were no significant differences between groups in *_m_d’* or *_m_*RT after performance was corrected by MoCA. (**A**,**B**) First 0-back condition (0b_1), Second 0-back condition (0b_2), 1-back condition (1b), 2-back condition (2b). (**A**,**B**) A 2-way ANOVA was used with Bonferroni multiple comparisons test to determine group and task effects. (**A**,**B**) Violin plots with solid and dotted lines are presented as median and interquartile range, respectively.

**Figure A3:**
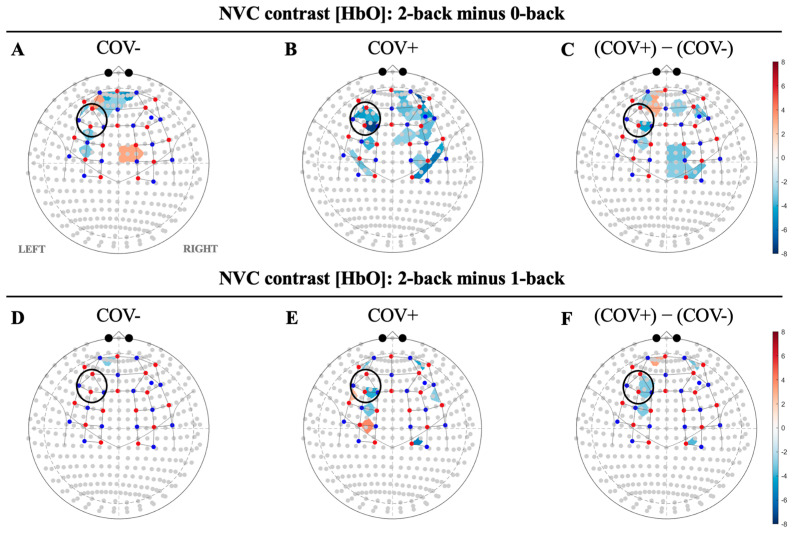
**NVC is decreased in LDLPFC in COV+ patients when corrected for age, sex, and education.** NVC responses were corrected for age, sex, and education. The black circle represents the left dorsolateral prefrontal cortex (LDLPFC), and red and blue shaded areas represent significant increases and decreases in [HbO], respectively. Within-group statistical comparison shows decreased LDLPFC NVC for COV+ in both contrasts assessing the most cognitively challenging task compared to less demanding tasks (**B**,**E**). The COV− group showed decreased LDLPFC NVC responses in 2b minus 0b (**A**) and no change in 2b minus 1b (**D**). Between-group comparison determined that COV+ NVC responses were decreased in the LDLPFC during the most cognitively challenging task relative to tasks that were less difficult compared to the COV− group. (**C**,**F**). Colors refer to t-statistics thresholded by *q* < 0.05 (obtained after false discovery rate correction).

To determine the fluid cognitive performance of the two groups, the *n*-back test performance was analyzed to determine MoCA-corrected and -uncorrected accuracy (*_m_d’* and *d’*, respectively), covering cognitive domains of attention, inhibition, and working memory, and reaction time (*_m_*RT and RT), as well as covering processing speed (uncorrected scores are presented in Figure A2). Accuracy reflects participants correctly clicking the mouse button when the target letter appeared, while reaction time measures the duration between the presentation of the target stimulus and the participant’s response. MCI has prominent dysfunction within these fluid cognitive domains, irrespective of etiology [55]. Moreover, as we age, impairments in executive functions and other fluid domains are hallmarks of cognitive aging [56,57], with progressive decline along the cognitive impairment continuum (e.g., MCI and dementia). There was no difference in cognitive performance between the COV+ and COV− groups for *_m_d’* (Figure 2A) and *_m_*RT (Figure 2B). There was a significant group effect of increased *d’* in COV+ compared to COV− when MoCA was not corrected (F (1, 39) = 5.632, *p* = 0.0227) and no difference in RT. Irrespective of COVID-19 status, the participants with MCI had significantly worse *d’* (F (2.454, 95.70) = 34.21, *p* < 0.0001) and increased RT (F (1.813, 70.09) = 23.23, *p* < 0.0001) during more challenging tasks (Figure A3).

### 3.4. Endothelial Function Is Impaired in MCI Participants with a History of COVID-19

**Figure 3:**
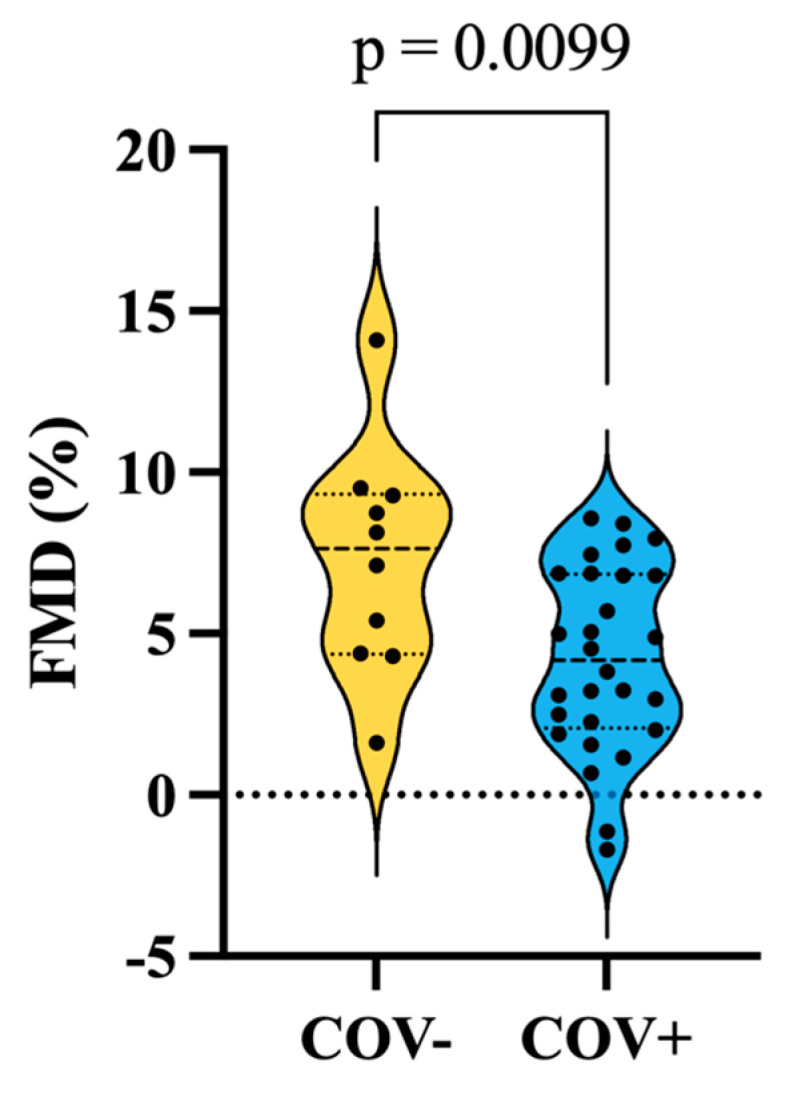
Endothelial function is impaired in MCI participants with a history of COVID-19. Maximal flow mediated dilation respective to baseline brachial artery (FMD%) diameter was significantly decreased in the COV+ group compared to the COV− group. Unpaired *t*-test was used to determine group differences in FMD%.

To determine systemic endothelial function, we employed the flow-mediated dilation protocol during insonation of the brachial artery. COV+ participants had significantly decreased maximal dilation during insonation of the brachial artery (FMD%) compared to COV− participants (COV+, 4.218 ± 2.850, COV−, 7.257 ± 3.513, *p* = 0.0099) (Figure 3). 

## 4. Discussion

In this study, we determined that a history of COVID-19 in patients with MCI affects the vasculature systemically, contributing to impaired NVC responses and decreased peripheral endothelial function in patients with MCI. Importantly, the NVC impairment was localized to the LDLPFC, which is an area largely responsible for working memory and executive function. This finding provides mechanistic insight into how COVID-19 may cause impairment, highlighting NVC’s critical role in this process and extending beyond the widely documented cognitive dysfunction associated with the viral infection.

COVID-19 has systemic effects on the endothelium, precipitating multi-organ dysfunction [58]. In vivo, in vitro, and histopathological evidence from preclinical models [59,60] and humans [24,34,61] show strong evidence of COVID-19-induced deleterious effects on endothelial function. Our findings of endothelial nitric oxide synthase [62] dependent vasodilatory dysfunction confirmed generalized endothelial impairment in the COV+ group compared to COV−. Due to the strong associations between peripheral vascular health and cognition [42], these data may find predictive value in the worsening of cognitive function in our future longitudinal analysis.

In the brain, evidence suggests that COVID-19 disrupts the integrity of the blood–brain barrier (BBB) [63], perpetuates CSVD pathology [38], contributes to cerebral atrophy [64], and impairs cerebral blood flow (CBF) regulation [65]. Importantly, one of the earliest predictors of cognitive impairment is decreased CBF [66]. Moreover, BBB disruption has been shown to worsen with increased clinical dementia rating [67]. These subclinical vascular disruptions may be present in healthy aging before any signs of cognitive dysfunction [66,68]. In our study, we demonstrated significantly impaired NVC responses in COV+ compared to COV− patients despite observing no measurable difference in MoCA-corrected cognitive dysfunction. As NVC plays a critical role in microvascular CBF regulation [69] and cerebral hypoperfusion is associated with accelerated cognitive decline [70] and a significant predictor of future cognitive impairment, the uncoupling of NVC shown here may predict future cognitive decrement associated with COVID-19. Previous work has indicated that COVID-19 accelerated the incidence of MCI progression to dementia over 18 months [20]. Hence, our hypothesis is that COVID-19-induced NVC impairment, particularly in the DLPFC, may precede cognitive deficits and contribute to the conversion rate from MCI to dementia, but this cross-sectional study design limits our ability to make causal assumptions about cognitive outcomes. Moreover, as diffuse cerebrovascular impairment—reflected by global decreases in cerebral blood flow—is associated with cognitive impairment, we will assess both local (LDLPFC) and global NVC impairments in the longitudinal arm of the current study.

This study was limited by unbalanced groups. During the recruitment for this study, the CDC estimated that >70% of individuals in the United States have had a SARS-CoV-2 infection. This limited our ability to enroll participants who were COVID-19-negative. However, due to our sample having a similar population estimated prevalence of cases and controls (COV+, N = 31 (74%); COV−, N = 11 (26%)), this lends increased generalizability to the study. Our study was strengthened by anti-NP testing to confirm or negate self-report data. While this is the most sensitive measure of determining past infection, it is limited in detecting a history of SARS-CoV-2 infection one year past infection [71]. This limitation may have resulted in patients who had an early infection that was asymptomatic being classified as COVID-19-negative.

## 5. Conclusions

In conclusion, our cross-sectional study, which is part of a prospective longitudinal analysis, found that patients with MCI and a history of COVID-19 exhibited impaired NVC and endothelial function compared to those without a history of COVID-19. These data are the first to indicate the effects of COVID-19 on the NVC response in the vulnerable patient population of MCI. The current findings, along with our consistent clinical and preclinical evidence, highlight the urgent need for further research and clinical trials to explore therapeutic interventions aimed at preserving and even restoring NVC. Pharmacological therapies that improve endothelial function, such as antioxidants, statins, ACE inhibitors/ARBs, and metformin, have shown promise in addressing long-term vascular dysfunction induced by COVID-19. In addition, lifestyle interventions, such as time-restricted eating and advanced strategies like transcranial magnetic brain stimulation, may offer further potential for mitigating neurovascular uncoupling (NVC) and cognitive impairment in this context [12,58]. While these approaches are promising, further research is essential to fully understand their mechanisms and therapeutic potential. Preventing the deterioration of cognitive function through targeted NVC therapies could delay progression to dementia from MCI, significantly enhance the quality of life for patients and their caregivers, and potentially reduce the huge economic burden associated with dementia care.

## Figures and Tables

**Table 1:** Demographics, conditions, medications, and baseline physiology of the study participants.

*Characteristics*	COV− (N = 11)		COV+ (N = 31)	
	*n*	%	*n*	%
**Sex**				
*Male*	7	16.67	23	54.76
*Female*	4	9.52	8	19.05
**Race**				
*American Indian or Alaska native*	0	0.0	0	0.0
*Asian*	0	0.0	0	0.0
*Black or African American*	0	0.0	0	0.0
*Native Hawaiian or Pacific Islander*	0	0.0	1	2.38
*White or Caucasian*	11	26.19	28	66.66
*Other*	0	0	2	4.76
**Ethnicity**				
*Hispanic/Latino*	0	0.0	1	2.38
*non-Hispanic/non-Latino*	11	26.19	30	71.43
**Highest level of education**				
*Doctorate degree*	2	4.76	5	11.90
*Master’s degree*	1	2.38	3	7.14
*Bachelor’s degree*	2	4.76	11	26.19
*Some college (<4 years)*	4	9.52	10	23.81
*Highschool/GED*	2	4.76	2	4.76
**Active cardiovascular diseases**				
*Hypertension*	8	19.05	18	42.86
*Dyslipidemia*	3	7.14	15	35.71
*Type 2 diabetes mellitus*	2	4.76	6	14.29
*Coronary artery disease*	3	7.14	4	9.52
*Congestive heart failure*	1	2.38	1	2.38
*Atrial fibrillation*	0	0.00	3	7.14
**Psychiatric diseases**				
*Depression*	6	14.29	11	26.19
*Anxiety*	5	11.90	11	26.90
*Post-traumatic stress disorder*	1	2.38	3	7.14
**Cerebrovascular accidents**				
*Stroke*	2	4.76	0	0.00
*Transient ischemic attack*	1	2.38	1	2.38
*Current cigarette smoker*	0	0.00	0	0.00
***Baseline data***	**Mean**	**S.D.**	**Mean**	**S.D.**
*Age [years]* *Systolic blood pressure [mmHg]*	73.18 135.40	5.76 18.61	74.16 141.10	4.82 13.32
*Diastolic blood pressure [mmHg]*	80.70	10.40	84.73	10.98
*Mean arterial blood pressure [mmHg]*	98.94	12.35	103.50	10.25
*Heart rate [beats per minute]*	69.12	11.59	68.22	12.46
*Body mass index [kg/m^2^]*	26.94	6.22	27.99	4.56
*Year of MCI diagnosis*	2022	1.80	2020	4.32
*Montreal Cognitive Assessment*	22.55	2.30	24.35	2.54

Ongoing medical conditions were pharmacologically controlled. Other conditions: gastroesophageal reflux disease (19), osteoarthritis (16), sleep apnea (9), dizziness (9), tremors (8), peripheral neuropathy (7), irritable bowel syndrome (4), asthma (3), migraine (3), chronic kidney disease (3), hypothyroidism (3), asthma (3), migraines (3), rheumatoid arthritis (3), restless leg syndrome (2), urinary incontinence (2), carpal tunnel (2), osteoporosis (2), diverticulosis (2), degenerative joint disease (2), chronic iron deficiency anemia (1), fibromyalgia (1), chronic obstructive pulmonary disorder (1), chronic pancreatitis (1), and trigeminal neuralgia (1).

## Data Availability

Custom codes for neurovascular coupling will be made available upon request. All values from graphed data are available in the Supporting Data Values file.
